# Gastrointestinal side effects in hepatocellular carcinoma patients receiving transarterial chemoembolization: a meta-analysis of 81 studies and 9495 patients

**DOI:** 10.1177/17588359251316663

**Published:** 2025-02-07

**Authors:** Nathalie Arendt, Maria Kopsida, Jaafar Khaled, Markus Sjöblom, Femke Heindryckx

**Affiliations:** Department of Medical Cell Biology, Uppsala University, Uppsala, Sweden; Department of Medical Cell Biology, Uppsala University, Uppsala, Sweden; Department of Medical Cell Biology, Uppsala University, Uppsala, Sweden; Department of Medical Cell Biology, Uppsala University, Uppsala, Sweden; Department of Medical Cell Biology, Uppsala University, Husargatan 3, Uppsala 75431, Sweden

**Keywords:** abdominal pain, chemotherapy agents, diarrhea, gastrointestinal side effects, GI toxicity, hepatocellular carcinoma, nausea, transarterial chemoembolization

## Abstract

**Background:**

Transarterial chemoembolization (TACE) is a widely used treatment for hepatocellular carcinoma (HCC), combining targeted chemotherapy and embolization. While effective, TACE can be associated with significant gastrointestinal (GI) side effects, impacting a patient’s quality of life.

**Objectives:**

Quantify the prevalence of key GI complications (diarrhea, nausea, GI toxicity, abdominal pain) following TACE.

**Design:**

Systematic review was performed following Preferred Reporting Items for Systematic reviews and Meta-Analyses (PRISMA) guidelines, focusing on studies that reported side effects of TACE. Studies not involving cTACE or drug-eluting bead TACE (DEB-TACE), non-HCC studies, meta-analyses or systematic reviews, and inaccessible publications were excluded.

**Data sources and methods:**

A PubMed search for clinical and randomized trials was conducted. Extracted data included study identifiers, demographics, TACE details, and GI side effect prevalences. The Mixed Methods Appraisal Tool assessed study quality and bias.

**Results:**

The analysis included data from 81 studies with 121 individual study arms and 9495 patients. Diarrhea was reported in 38 studies, with a mean prevalence of 23.46% (2.5; 95% confidence interval (CI): 18.39–28.544) and a weighted prevalence of 23.5%. Nausea was most frequently reported, mentioned in 67 studies, with a mean prevalence of 34.66% (2.4; 95% CI: 29.89–39.44) and a weighted prevalence of 32.5%. Abdominal pain was reported in 59 studies, with the highest mean prevalence of 48.07% (2.9; 95% CI: 42.20–53.93) and a weighted prevalence of 46.1%. GI toxicity was reported in 32 studies, with a mean prevalence of 8.85% (1.4; 95% CI: 5.99–11.70) and a weighted prevalence of 9.9%. DEB-TACE generally led to slightly higher rates of nausea, diarrhea, abdominal pain, and GI toxicity compared to conventional TACE. The type of chemotherapy agent influenced prevalence of GI-side effects, with high prevalences observed for agents such as zinostatin and cisplatin.

**Conclusion:**

This meta-analysis synthesizes current evidence on managing GI side effects in TACE. Standardizing reporting and developing effective management strategies are crucial to improving patient outcomes.

## Plain language summary

Gastrointestinal issues from a liver cancer treatment: what patients and doctors should know

Transarterial Chemoembolization (TACE) is a treatment used for liver cancer that combines chemotherapy with a procedure to block the blood supply to the tumor. While TACE can be effective, it often causes gastrointestinal (GI) side effects, which can impact the patient’s quality of life and ability to continue treatment.

This summary explains the findings of a study that looked at the prevalence and types of GI side effects experienced by patients undergoing TACE.

The study analyzed data from 81 clinical trials, involving a total of 9495 patients. Researchers looked at the most common GI side effects, like nausea, abdominal pain, diarrhea, and GI toxicity (which includes issues like ulcers and bleeding).

1. Nausea: This was the most frequently reported side effect, affecting about 34.7% of patients. Nausea can reduce appetite and overall well-being.

2. Abdominal Pain: Reported by 48.1% of patients, abdominal pain was the most prevalent symptom. This pain can be due to the procedure itself or the chemotherapy drugs used.

3. Diarrhea: Around 23.5% of patients experienced diarrhea, which can lead to dehydration and nutritional issues.

4. GI Toxicity: This included a range of severe digestive problems and affected about 8.9% of patients.

The study also found that the type of TACE and the specific chemotherapy drugs used can influence the severity of these side effects. For example, a newer method called DEB-TACE, which uses drug-eluting beads, tends to cause more nausea and abdominal pain compared to conventional TACE. Additionally, certain drugs, like zinostatin and cisplatin, were linked to higher rates of nausea and abdominal pain.

Understanding these side effects is important for managing them effectively and improving patient care. The study suggests the need for standardized reporting of side effects in future research to better assess risks and develop strategies to minimize discomfort for patients.

## Introduction

Transarterial chemoembolization (TACE) is a widely used locoregional therapy for hepatocellular carcinoma (HCC), particularly among patients who are not candidates for surgical resection or liver transplantation.^1–3^ By delivering high concentrations of chemotherapeutic agents directly into the liver tumor and concurrently obstructing the tumor’s arterial blood supply, TACE aims to induce cancer cell death while preserving surrounding liver tissue.^4^ There are two principal forms of TACE: conventional TACE (cTACE) and drug-eluting bead TACE (DEB-TACE).^5^ cTACE uses an emulsion of chemotherapeutic agents with an oily contrast medium,^4^ while DEB-TACE uses microspheres that slowly release the chemotherapeutic agent over time, potentially reducing systemic exposure and associated adverse effects (AE).

Despite its therapeutic benefits, TACE is associated with a range of potential complications and AE, with gastrointestinal (GI) disturbances being particularly prevalent.^6–8^ These disturbances are multifactorial and commonly include symptoms such as diarrhea, nausea, oral mucositis, and abdominal pain.^9^ Notably, many of these GI symptoms are encompassed under the umbrella of postembolization syndrome (PES), which is one of the most common AEs of TACE. PES occurs in a significant proportion of patients—with incidence rates reported to range from 30% to 90%^10^—and typically develops within 1–2 weeks after the procedure and includes symptoms such as nausea, vomiting, anorexia, fever, and abdominal pain. The overlap between GI disturbances and PES highlights that these symptoms are significant components of the syndrome. Although the exact mechanisms are not fully understood, systemic effects of chemotherapeutic agents, inflammatory responses resulting from ischemia, and necrosis of tumor cells are believed to contribute to PES and can explain some of the GI complications.^11–13^ The high prevalence underscores the need for vigilant postoperative monitoring and supportive care to mitigate symptoms, prevent treatment delays, and improve patients’ quality of life.^14,15^

Various factors influence the prevalence and severity of PES and specific GI complications, such as the type and dose of chemotherapeutic agents, the technique used, and individual patient characteristics, such as liver function and comorbidities.^16–18^ There is currently no standard dosage of chemotherapy in TACE procedures (doxorubicin dosing ranging from 30 to 75 mg/m^2^ up to a maximum of 150 mg/m^2^), and many different chemotherapeutics are used either in the TACE-formulation or in combination with TACE.^19–21^ This variation in clinical practice likely contributes to different GI complications that can severely impact the quality of life. Diarrhea is particularly concerning due to its potential to cause dehydration, electrolyte imbalances, and malnutrition, which can compromise patient outcomes and disrupt treatment schedules.^22^ Nausea and intestinal mucositis can also reduce patients’ nutritional intake and overall well-being.^17,18,23^ Cumulative rates of nausea and vomiting following TACE have been estimated at 44.8% and 27.6%, respectively,^24^ thus potentially affecting a large group of patients with intermediate-stage HCC. Abdominal pain, a more direct indicator of local toxicity, can signal serious underlying GI complications or result from the ischemia induced by the embolization process in hepatic tissue.^11^ Additionally, chemotherapy-induced intestinal toxicity can exacerbate the abdominal discomfort experienced by patients undergoing TACE,^13^ highlighting the need for comprehensive management strategies that address both procedural effects and systemic toxicity.^8,11^

Despite the clinical significance of these symptoms, the literature on their prevalence, management, and impact on patient outcomes in the context of TACE remains fragmented and inconsistent. The heterogeneity in reporting AE across different studies complicates the ability to draw definitive conclusions regarding the GI toxicity profiles of various TACE regimens. Selective reporting and differing research priorities contribute to inconsistencies in documenting these side effects, thereby limiting the generalizability of findings and hindering efforts to optimize patient care.^25^ Standardizing the reporting of AE in TACE studies is essential to accurately assess the risk-benefit ratio of different treatment modalities and to develop effective management strategies.^6^ The primary aim of this systematic review is to thoroughly investigate the prevalence of GI complications in patients who have undergone TACE for HCC, with a particular focus on diarrhea, nausea, GI toxicity (encompassing mucositis, GI hemorrhaging, and ulcerations), and abdominal pain. By providing a comprehensive synthesis of existing research, this review seeks to identify gaps in knowledge, offer insights into the pathophysiology of TACE-related GI toxicity, and guide clinical practices to improve patient care and outcomes.

## Methods

### Eligibility criteria

**Figure 1.**
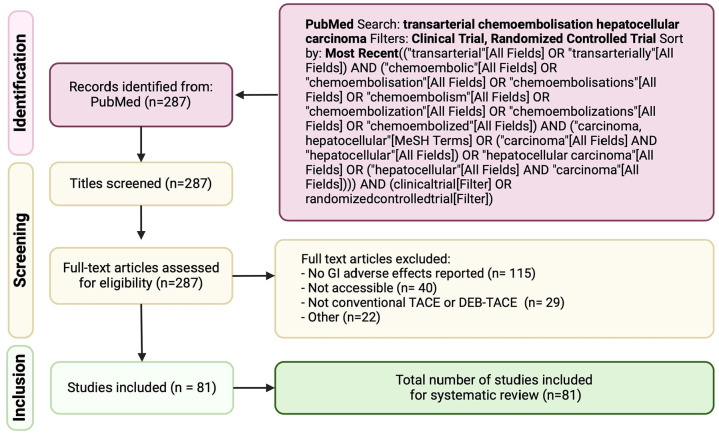
PRISMA diagram depicting the flow of information through the different phases of the systematic review. PRISMA, Preferred Reporting Items for Systematic Reviews and Meta-Analyses.

To be included in this review, studies had to report on the GI side effects subsequent to TACE treatment, with explicit data on diarrhea, nausea, GI toxicity (defined as GI mucositis, ulcerations, and/or hemorrhaging), and abdominal pain. The literature search was confined to clinical trials and randomized controlled trials (RCTs) to ensure the inclusion of studies with robust experimental designs. Excluded from this review are studies that did not specify GI side effects or were unrelated to TACE, as well as meta-analyses, systematic reviews, case reports, and inaccessible publications. The Preferred Reporting Items for Systematic reviews and Meta-Analyses (PRISMA) were followed during this study (Supplemental File 1 and Figure 1). The review protocol was not registered.

### Information sources and search strategy

The literature search was executed across PubMed. The search terms were centered around “transarterial chemoembolization” combined with “hepatocellular carcinoma,” with the application of filters to isolate clinical trials and RCTs (Supplemental File 2). The sorting of search results prioritized the most recent studies. Data collection was completed by four independent researchers. All review methods were established prior to the conduct of the data collection.

### Data collection

The data extraction involved the study identifier (PMID), author, publication year, and study design, along with the geographical location where the research was conducted. Participant demographics were cataloged, detailing the sample size and the median age of the subjects. Details regarding the TACE procedure were collated, including the type of TACE performed, the chemotherapeutic agents utilized, and any combination treatments administered. The primary outcomes of this systematic review were the prevalence of the specified GI AE, including diarrhea, nausea, abdominal pain, and GI toxicity (including mucositis, ulcerations, and GI hemorrhages) in different TACE treatments, which were included as percentages. Side effects that were not measured or reported in a study were treated as missing values rather than zeros. Missing values were excluded from the calculation of prevalence to ensure the accuracy of the data representation and reduce the risk of bias due to missing results. The quality and bias of studies were assessed using the mixed methods appraisal tool (MMAT), performed by three independent researchers.^26^ Inconsistencies were double-checked by a fourth independent, senior researcher. Results are presented as ratings for each criterion (Supplemental Table 3), as per MMAT guidelines.^26^

### Standardization of side effects

To normalize the varying baseline prevalence rates of different side effects, each side effect score was standardized to ensure equal contribution to the overall prevalence score, forming the GI-adverse effect (GI-AE) scoring system. First, the mean and standard deviation were calculated for each side effect across all studies reporting that specific side effect. Each prevalence value was then standardized using the formula:

$$z=\frac{x-\mu }{\sigma }$$

where *z* is the standardized score, *x* is the original prevalence value, *μ* is the mean prevalence, and σ is the standard deviation. This transformation resulted in a mean of 0 and a standard deviation of 1 for each side effect. For each study, the overall standardized prevalence score, termed the GI-AE score, was computed by summing the standardized scores of the reported side effects.

### Statistical analyses

Data was collected in Excel and imported in GraphPad Prism version 10.2.3 (GraphPad Software, Boston, Massachusetts, USA). Bar charts were plotted representing mean prevalences within the included studies, with error bars representing standard error-of-the-mean (SEM). To explore possible causes of heterogeneity among study results, subgroup analyses were performed, using different treatment regimens as subgroups. Unpaired Student’s *t*-test was performed to assess statistical significance. For multivariate unsupervised analysis, we used principal component analysis (PCA) after scaling the data to have a mean of 0 and a standard deviation of 1.^27,28^ Principle components were then selected based on eigenvalues using the Kaiser rule.

## Results

### Study selection

Our systematic search across PubMed was conducted to identify studies assessing GI side effects in HCC-patients undergoing TACE treatment. The search strategy was designed with a combination of keywords and MeSH terms related to “TACE,” “Hepatocellular carcinoma,” and similar terms, spanning literature from 1990 to 2023. We applied filters to only include clinical trials and RCTs, thereby excluding systematic reviews, meta-analyses, and case reports.

After the initial search, a total of 287 records were identified (Supplemental Table 4). All records underwent title and abstract screening, applying our predetermined inclusion and exclusion criteria. Criteria for exclusion included studies outside the scope of TACE treatment, pediatric studies, and those not addressing any GI side effects. Upon full-text review of these articles, 115 studies were excluded due to a lack of direct relevance to GI side effects, 40 studies were inaccessible, 29 did not use cTACE or DEB-TACE, and 22 were excluded for other reasons (Supplemental Table 5), including study retraction, or not studying HCC or not meeting the criteria in terms of the type of study, leaving a total of 81 studies to be included in our analyses (Table 1). The quality of studies was assessed using the MMAT tool, and a detailed rating of each criterion is provided for each study (Supplemental Table 3).

**Table 1.** Summary of study characteristics, treatment modalities, and gastrointestinal AE reported in included studies evaluating TACE for hepatocellular carcinoma.

Author	Year	Country	*N*	Age	TACE	Chemotherapy	Adjuvant	Diarrhea (%)	Nausea (%)	Abdominal Pain (%)	GI toxicity (%)
Li^29^	2023	China	87	56	cTACE	Multiple	TKI and camrelizumab		33.3	62.1	
Bush^30^	2023	USA	39	59.6	cTACE	Multiple		2.6	25.0	58.9	5.2
Simasingha^31^	2023	Inda	50		cTACE	Mitomycin	Dexametasone		2.0	2.0	
Simasingha^31^	2023	Inda	50	60.46	cTACE	Mitomycin			64.0	38	
Shi^32^	2023	China	45	58.9	DEB-TACE	Pirarubicin			22.2	93.3	
Shi^32^	2023	China	45	60.6	cTACE	Pirarubicin			26.7	95.6	
Guo^33^	2023	China	61	58	DEB-TACE	Epirubicin	Sintilimab	8.0	36.0		21.0
Chiang^34^	2023	China	33	68	cTACE	Cisplatin	Radiation + avelumab	12.0	45.0	21.0	15.0
Comito^35^	2022	Italy	19	75	cTACE	Epirubicin			0.0	3.0	
Peng^36^	2022	China	170	54	Both	Multiple	Lenvatinib	47.1	35.9	50.6	
Chen^37^	2022	Taiwan	25	63	Both	Not specified		8.0			
Chen^37^	2022	Taiwan	29	66.6	Both	Not specified	Sorafenib	20.7			6.9
Li^38^	2022	China	155	54	cTACE	Epirubicin		4.0	43.0	52	
Aramaki^39^	2022	Japan	219	71	cTACE	Cisplatin			9.1	6.4	
Aramaki^39^	2022	Japan	213	70	cTACE	Epirubicin			8.0	6.6	
Fu^40^	2021	China	80		cTACE	Bleomycin			35.0	50	
Fu^40^	2021	China	80		cTACE	Doxorubicin		2.5	38.8	56.3	
Li^41^	2021	China	172		DEB-TACE	Doxorubicin			50.6	40.7	
Ding^42^	2021	China	32	56	DEB-TACE	Epirubicin	Lenvatinib	40.6	65.6	71.9	3.1
Ding^42^	2021	China	32	57	DEB-TACE	Epirubicin	Sorafenib	31.3	50.0	43.6	9.4
Zaltou^43^	2021	Irak	84	51.3	cTACE	Doxorubicin			6.0	28.6	
Zaitoun^43^	2021	Irak	89	52.1	cTACE	Doxorubicin	Microwave ablation		4.5	16.9	
Gjoreski^44^	2021	North macedonia	28	67.9	cTACE	Doxorubicin			67.9		
Gjoreski^44^	2021	North macedonia	32	68.8	DEB-TACE	Doxorubicin			62.5		
Guo^45^	2021	China	60	59.1	cTACE	Epirubicin	Oxaliplatin		48.3	83.4	
Guo^45^	2020	China	55	56.3	cTACE	Epirubicin	Oxaliplatin + S-1		43.6	85.5	
Turpin^46^	2021	France	39	66	DEB-TACE	Doxorubicin	Sunitinib	5.1		10.3	2.6
Turpin^46^	2021	France	39	67.4	DEB-TACE	Doxorubicin		0		7.9	2.6
Kudo^47^	2020	Japan	77	72	cTACE	Multiple	Sorafenib	14.3			
Kudo^47^	2020	Japan	71	73	cTACE	Multiple					
Sun^48^	2020	China	275	58.7	DEB-TACE	Multiple			12.6	55.0	
Park^49^	2019	South Korea	170	60.2	cTACE	Doxorubicin	Sorafenib	39.2	37.9	53.6	
Zhou^50^	2018	China	99	57.98	DEB-TACE	Multiple			16.1	96.0	
Xu^51^	2018	China	51	60	cTACE	Cisplatin	Sunitinib	41.2	39.2	69.0	2.0
Xu^51^	2018	China	53	62	cTACE	Cisplatin	Sorafenib	45.0	47.0	72.0	2.0
Yoon^52^	2018	South Korea	45	55	cTACE	Cisplatin	Radiation	8.8	71.1	80.0	0.0
Wang^53^	2018	China	140	54.2	Both	Not specified			48.6	17.1	
Zhang^54^	2018	China	37		cTACE	Epirubicin	Brachytherapy	70.3			
Zhang^54^	2018	China	31		cTACE	Epirubicin	Sorafenib	61.3			25.8
He^55^	2017	China	41		cTACE	Epirubicin		2.44	43.9	65.9	4.9
Goda^56^	2017	Japan	57	74	cTACE	Epirubicin			6.0	17.0	
Goda^56^	2017	Japan	18	71	cTACE	Miriplatin			17.0	23.0	
Yang^57^	2017	South Korea	41		cTACE	Doxorubicin			82.5	75.0	
Yang^57^	2017	South Korea	40		cTACE	Doxorubicin	Dexametasone		53.7	53.7	
Ikeda^58^	2018	Japan	124	72	cTACE	Miriplatin			44.4	64.5	
Ikeda^58^	2018	Japan	123	71	cTACE	Epirubicin			54.5	76.4	
Zhao^59^	2017	China	31	64.9	cTACE	Not specified	Lobaplatin + Huaier granule	32.3		16.1	
Zhao^59^	2017	China	31	62.7	cTACE	Not specified	Lobaplatin	25.8		22.6	
Meyer^60^	2017	UK	157	65	DEB-TACE	Doxorubicin	Sorafenib	56	46.0	59.0	41.0
Meyer^60^	2017	UK	156	68	DEB-TACE	Doxorubicin		31	43.0	57.0	18.0
Chan^61^	2017	Hong Kong	50	61.8	cTACE	Not specified	Axitinib	40	42.0	75.0	
Tong^62^	2017	China	35	55.4	cTACE	Doxorubicin		9	26.0	34.0	0.0
Tong^62^	2017	China	36	56.7	cTACE	Doxorubicin	Celecoxib + lanreotide	14.0	8.3	14.0	3.0
Zhao^63^	2016	China	76	55	cTACE	Raltitrexed	Oxaliplatin	9.2	33.0	43.4	
Zhao^63^	2016	China	76	54	cTACE	5-Fu	Oxaliplatin	11.8	31.6	55.4	1.3
Zhao^63^	2016	China	75	55	cTACE	Doxorubicin	Oxaliplatin	5.3	20.0	61.4	1.3
Yao^64^	2016	China	50	56.5	cTACE	Not specified	Sorafenib	38.0			14.0
Lencioni^65^	2016	USA	154	64.5	DEB-TACE	Doxorubicin	Sorafenib	52.9	37.9	60.1	20.9
Lencioni^65^	2016	USA	153	63	DEB-TACE	Doxorubicin		17.2	39.1	61.6	14.6
Ma^66^	2015	China	173	52.19	cTACE	Not specified	Licartin		57.5	68.8	2.4
Wang^67^	2015	China	61	54.75	cTACE	Multiple	Arsenic trioxide	4.92	6.6		
Wang^67^	2015	China	64	55.39	cTACE	Multiple		1.56	10.9		
Hoffman^9^	2015	Germany	24	58.5	cTACE	Carboplatin	Sorafenib	37.5	12.5		
Hoffman^9^	2015	Germany	26	58	cTACE	Carboplatin		12.0	8.0		
Liu^68^	2015	China	50	54.2	cTACE	Doxorubicin			100	100	
Liu^68^	2015	China	59	53.5	cTACE	Multiple			100	100	
Liu^68^	2015	China	53	52.5	cTACE	Multiple			100	100	
Liu^68^	2015	China	70	55	cTACE	Not specified	Arsenic trioxide	7.1	10.0		
Choi^69^	2014	Korea	31	63.2	cTACE	Multiple	Radiation		45.2	12.9	
Chao^70^	2015	Taiwan	192	56.5	cTACE	Doxorubicin	Sorafenib	31.3	25.0	52.6	5.2
El Fouly^71^	2015	Germany + Egypt	42	58.3	cTACE	Adriamycin			38.0	83.0	0.0
Kudo^72^	2014	Japan	249	57	cTACE	Not specified	Brivanib	37.0	28.0	37.0	
Kudo^72^	2014	Japan	253	59	cTACE	Not specified		10.0	27.0	40.0	
Boulin^73^	2014	France	21	64	DEB-TACE	Idarubicin		5.0	14.0		5.0
Cho^74^	2014	Korea	67	55	cTACE	Adriamycin	Radiation		46.3	11.9	
Tzeng^75^	2008	Taiwan	98	60.6	cTACE	Epirubicin	Ionic contrast				2.04
Tzeng^75^	2008	Taiwan	99	62	cTACE	Epirubicin	Non-ionic contrast				1.01
Molinari^76^	2006	Canada	47	63.4	cTACE	Doxorubicin					3.7
Li^77^	2006	China	40	54.4	cTACE	Multiple	5-Fluorouracil		22.1		
Jang^78^	2004	Korea	34	56	cTACE	Cisplatin	Angiography		56.0	47	
Poon^79^	2004	China	41	59	cTACE	Cisplatin	Bcaa				2.4
Poon^79^	2004	China	43	59	cTACE	Cisplatin					7
Lo^80^	2002	Hong Kong	40	62	cTACE	Cisplatin			16.7	26	4.2
Kwok^81^	2000	Hong Kong	52	63	cTACE	Cisplatin	Gelfoam		39.1		4.3
Kwok^81^	2000	Hong Kong	48	65	cTACE	Cisplatin	Bloodclot		40.5		2.4
Chung^82^	2000	South Korea	19	29	cTACE	Cisplatin	IFN-alpha		53.0		11
Chung^82^	2000	South Korea	23	52	cTACE	Cisplatin					10
Inaba^83^	2013	Japan	50		cTACE	Epirubicin	Tsu-68	40		18	
Kasai^84^	2013	Japan	262		cTACE	Cisplatin			79.4	66.7	
Zhai^85^	2013	China	184		cTACE	Pirarubicin	Mitomycin C		6.3	15.8	
Meyer^86^	2013	UK	44	63.2	cTACE	Cisplatin			2.3	27.9	
Bai^87^	2013	China	82	54	cTACE	Doxorubicin	Sorafenib	36.6			7.3
Bai^87^	2013	China	222	50	cTACE	Doxorubicin					11
Iwazawa^88^	2012	Japan	48	72	cTACE	Miriplatin	Epirubicin		43.0	58	
Iwazawa^88^	2012	Japan	51	73	cTACE	Miriplatin			54.0	52	
Shi^89^	2013	China	122		cTACE	Multiple			23.6	48.3	
Shi^89^	2013	China	121		cTACE	Multiple			19.9	44.2	
Shi^89^	2013	China	122		cTACE	Epirubicin			20.5	51.1	
Chung^90^	2013	China	147		cTACE	Doxorubicin	Sorafenib	13.6	2.0	41.5	2.7
Park^91^	2013	Korea	30	51	cTACE	Adriamycin	Radiation + 5-FU	26.7	65.3		26.6
Morimoto^92^	2013	Japan	132	70	cTACE	Epirubicin	Radiation			6.8	
Morimoto^92^	2013	Japan	132	70	cTACE	Miriplatin	Radiation			6.8	
Kamimura^93^	2012	Japan	9	73.6	cTACE	Miriplatin	Ddp-h				
Osuga^94^	2012	Japan	46	64.6	cTACE	Cisplatin			58.7	37.0	
Sansonno^95^	2012	Italy	31	73	cTACE	Multiple	Sorafenib	10	17.5		
Sansonno^95^	2012	Italy	31	72.8	cTACE	Multiple		7.5	7.5		
Park^96^	2012	Korea	50	61.5	cTACE	Doxorubicin	Sorafenib	48.0	54.0	64.0	34
Britten^97^	2012	USA	15	61	cTACE	Multiple	Bevacizumab		40.0	73.3	
Britten^97^	2012	USA	14	58	cTACE	Multiple			64.3	92.9	
Sieghart^98^	2012	Austria	15	67	cTACE	Doxorubicin	Sorafenib	53.3	53.3	93.3	
Toyama^99^	2012	Japan	19	67.1	DEB-TACE	Cisplatin			10.5	52.6	
Vogl^13^	2012	Germany	102	67	DEB-TACE	Doxorubicin			4.6	5.8	5.4
Vogl^13^	2012	Germany	110	67.3	cTACE	Doxorubicin			4.1	21.5	7.4
Pawlik^100^	2011	USA	35	63	DEB-TACE	Doxorubicin	Sorafenib	39.0	11.0	6.0	6
Boulin^101^	2011	France	13		cTACE	Pirarubicin	Amiodarone		19.0	49.0	
Boulin^101^	2011	France	14		cTACE	Pirarubicin	Sorafenib		16.0	50.0	
Kudo^102^	2011	Korea + Japan	229	69	cTACE	Multiple		31.0			
Kudo^102^	2011	Korea + Japan	229	70	cTACE	Multiple		5.0	1.0		
Kim^103^	2011	Korea	31	55	cTACE	Doxorubicin					22.5
Reyes^104^	2009	USA	20	64	DEB-TACE	Doxorubicin			15.0	40.0	
Okusaka^105^	2009	Japan	79	65	cTACE	Zinostatin		3.0	54.0	70.0	

Columns list the first author, year of publication, and the country where the study was conducted, sample size (*N*), and median patient age. The TACE modality columns specify whether cTACE or DEB-TACE was used, along with details on the chemotherapy agents used within TACE. Any additional adjuvant therapies administered in conjunction with TACE are also noted. Reported GI-AE include prevalence of diarrhea, nausea, and abdominal pain, as well as the overall GI toxicity percentage. All AE are presented as percentages (%).

AE, adverse effect; cTACE, conventional TACE; DEB-TACE, drug-eluting bead TACE; GI, gastrointestinal; TACE, transarterial chemoembolization.

### Characteristics of included studies

**Figure 2.**
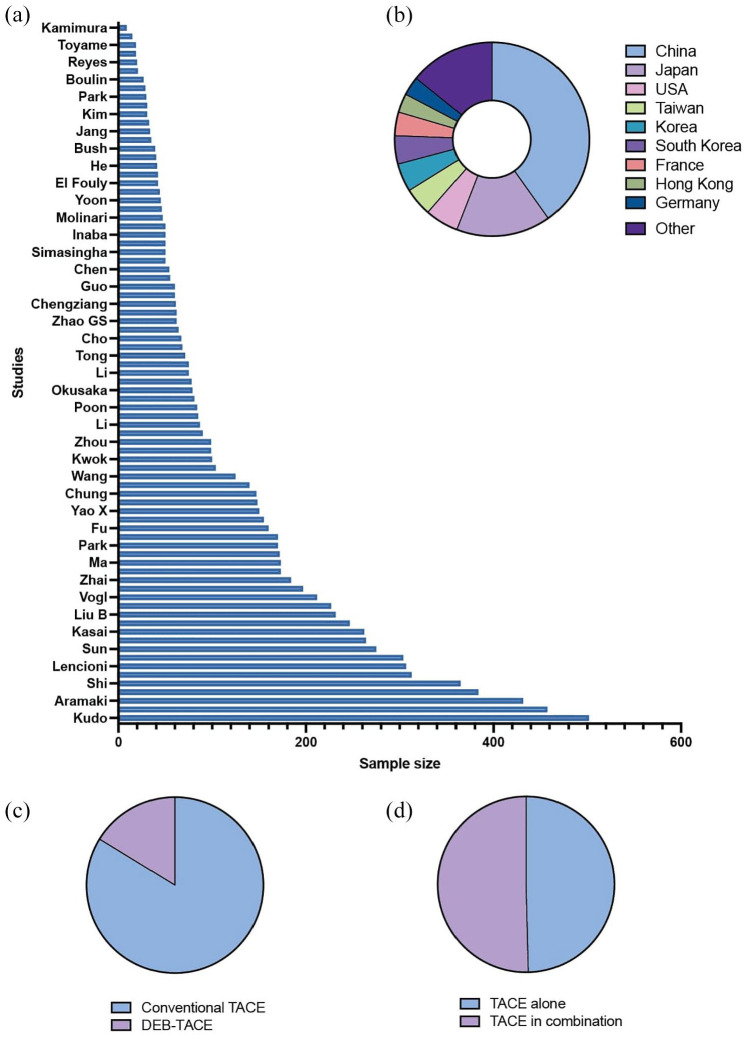
Overview of studies and treatment modalities. (a) Distribution of sample sizes across various studies investigating TACE. The bar chart displays the sample size for each study. (b) Geographic distribution of the studies, represented as a donut chart. The chart illustrates the proportion of studies conducted in various countries, with China, Japan, and the USA contributing the largest shares. (c) Proportion of studies using conventional TACE versus DEB-TACE. (d) Proportion of studies investigating TACE alone versus TACE in combination with adjuvant treatments. DEB-TACE, drug-eluting bead-transarterial chemoembolization.

The included studies varied in design, geographical location, and sample size (Table 1). They comprised 62 RCTs and 17 prospective cohort studies. Studies including several treatments were divided per treatment arm, in order to assess differences in TACE modalities. The total number of patients across all studies amounted to 9495, with study sizes ranging from 9 participants^93^ to 502 participants^72^ (Figure 2(a)). The geographical distribution of these studies spanned 20 countries worldwide, with the majority of studies conducted in China and Japan (Figure 2(b)). Notably, no studies have been included from Oceania or Africa.

Our systematic review encompassed different TACE treatment modalities across the included studies. Particular attention was paid to studies that investigated multiple treatment modalities within the same study, such as differences between DEB-TACE and cTACE (Figure 2(c)), variations in the emulsion used in TACE procedures, or the inclusion of adjuvant treatments (Figure 2(d)). Each treatment arm within these studies was analyzed as a separate entity (study arm), leading to a total of 121 study arms. In terms of TACE technique specificity, the majority of study arms reported the use of cTACE. Conversely, DEB-TACE was examined in 19 study arms, indicating a growing interest in this method that promises controlled drug release and reduced systemic toxicity.^5^ In 60 study arms, TACE was used in combination with additional treatments, whereas 61 study arms included TACE as a monotherapy. Notably, the combination of TACE with Sorafenib emerged as the most common combinational treatment strategy, represented in 18 study arms. The composition of the TACE emulsion—a critical factor influencing treatment efficacy and side effect profile—also varied among the study arms. Doxorubicin was the most frequently reported chemotherapeutic agent, used in 31 study arms. This was followed by epirubicin, reported in 18 study arms, cisplatin in 17, and miriplatin in 6, reflecting a wide range of clinical practices and preferences in the use TACE for HCC management.^19,20^ The least common chemotherapeutics used in the TACE-procedure within the included studies were 5-fluorouracil,^63^ bleomycin,^40^ idarubicin,^73^ raltitrexed,^63^ and zinostatin.^105^

### Prevalence and reporting of GI side-effects

**Figure 3.**
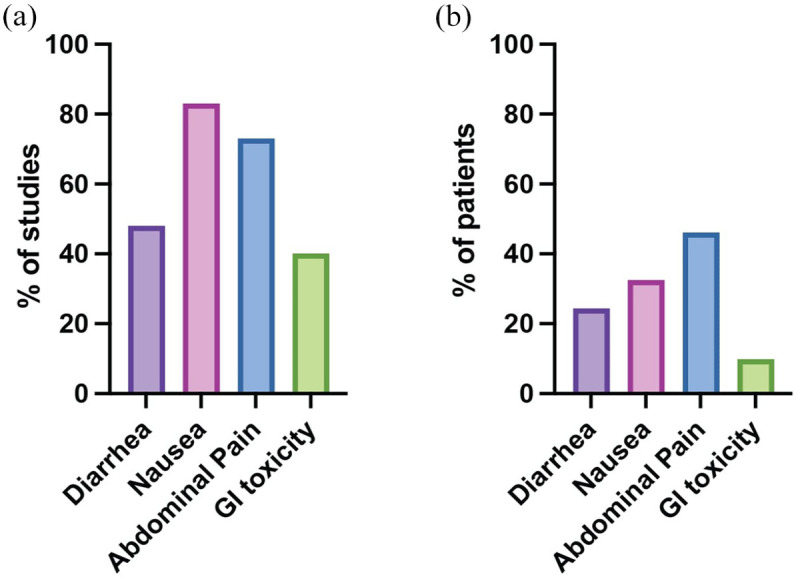
Documentation and prevalence of GI side effects. (a) Percentage of included studies reporting the different GI-related adverse effects. (b) Prevalence of symptoms within included studies. GI, gastrointestinal.

In our meta-analyses of 81 studies, we observed variability in the documentation of specific GI side effects, as detailed in Figure 3(a) and Table 2. Diarrhea was explicitly measured as a side effect in 48% of these studies. Similarly, nausea was reported in 83% of the studies and abdominal pain in 73% of the studies, while different forms of GI toxicity (including ulcerations, intestinal hemorrhages, and mucositis) were reported in 40% of the studies (Figure 3(a)). These figures represent the proportion of studies acknowledging the presence of each GI symptom in patients undergoing TACE, without suggesting the overall prevalence of these symptoms.

**Table 2.** Prevalence of gastrointestinal symptoms and toxicity in HCC-patients undergoing TACE.

Symptom	Number of studies (*n*)	Individual study arms (*n*)	Cases (*n*)	Mean prevalence (%)	Weighted prevalence (%)
Diarrhea	39	54	1046	23.46	24.4
Nausea	67	96	2536	34.66	32.5
Abdominal pain	59	86	3401	48.07	46.1
GI toxicity	32	45	312	8.85	9.9

HCC, hepatocellular carcinoma; TACE, transarterial chemoembolization.

We calculated the mean and weighted prevalence for these four GI-related side effects (Table 2 and Figure 3(b)). Diarrhea was reported in 38 studies, with a mean prevalence of 23.46% (2.5; 95% confidence interval (CI): 18.39–28.544) and a weighted prevalence of 23.5%. Nausea was the most frequently reported symptom, mentioned in 67 studies, with a mean prevalence of 34.66% (2.4; 95% CI: 29.89–39.44) and a weighted prevalence of 32.5%. Abdominal pain was reported in 59 studies, with the highest mean prevalence of 48.07% (2.9; 95% CI: 42.20–53.93) and a weighted prevalence of 46.1%. GI toxicity was reported in 32 studies, with a mean prevalence of 8.85% (1.4; 95% CI: 5.99–11.70) and a weighted prevalence of 9.9%.

### Diarrhea

**Figure 4.**
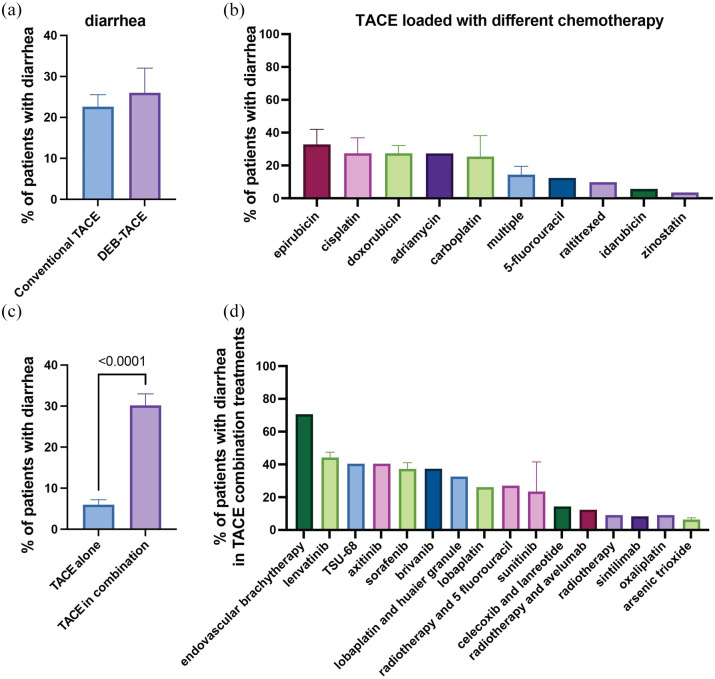
Prevalence of diarrhea in patients undergoing TACE with various treatment regimens. (a) Comparison of the percentage of patients experiencing diarrhea between those treated with conventional TACE and DEB-TACE. (b) Prevalence of diarrhea in patients treated with TACE loaded with different chemotherapeutic agents. The percentage of patients experiencing diarrhea varied across different chemotherapeutics, with doxorubicin, cisplatin, and epirubicin showing higher rates compared to other agents such as idarubicin and zinostatin. (c) Comparison of the prevalence of diarrhea in patients undergoing TACE alone versus TACE with adjuvant treatments. (d) Prevalence of diarrhea in patients treated with TACE in combination with various other treatments. Bar charts represent mean prevalence ± SEM. DEB-TACE, drug-eluting bead TACE; SEM, standard-error-of-the-mean; TACE, transarterial chemoembolization.

Our meta-analysis identified diarrhea as a reported side effect in 39 of the analyzed studies (Table 2). The mean prevalence of diarrhea across these studies was found to be 23.5% (Table 2 and Figure 3(b)). Notably, patients undergoing DEB-TACE exhibited a slightly higher prevalence of diarrhea compared to those receiving cTACE (Figure 4(a)). Furthermore, the prevalence of diarrhea varied depending on the type of chemotherapy used in the TACE emulsion (Figure 4(b)). Studies using epirubicin and cisplatin reported a mean prevalence of diarrhea in 32.2% and 26.7% of patients, respectively (Figure 4(b)). One study using zinostatin reported the lowest prevalence of diarrhea at 3% (Figure 4(b)).^105^ The prevalence of diarrhea was generally lower in patients who underwent TACE alone (Figure 4(c)). Combining TACE with adjuvant therapies significantly increased the prevalence of this side effect. The highest prevalence was observed in one study where 70.3% of patients who received TACE in combination with endovascular brachytherapy reported diarrhea as a side effect (Figure 4(d)).^106^ Alarmingly, studies combining TACE with sorafenib^37,42,49,51^ or TACE with lenvatinib^36,42^ reported a relatively high prevalence of diarrhea, with 36.94% and 43.85% of patients, respectively, reporting this side effect in the included studies. This is particularly concerning given the increasing use of these adjuvant treatments in clinical practice.^107,108^

### Nausea

**Figure 5.**
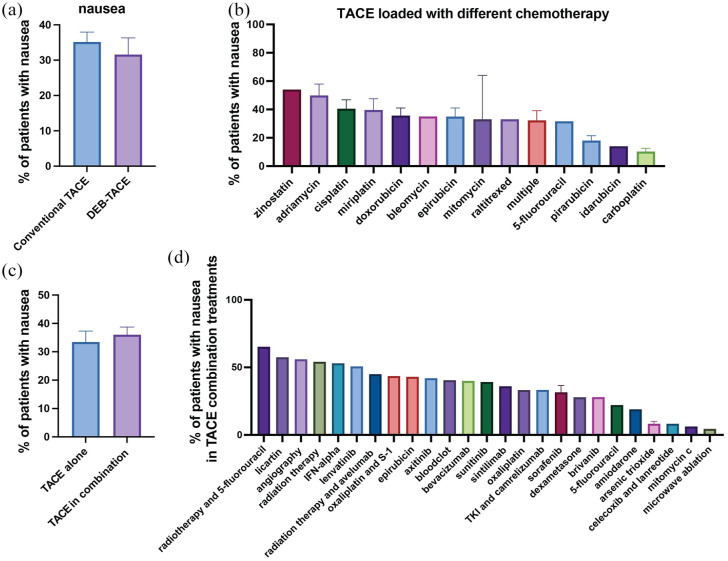
Prevalence of nausea in patients undergoing TACE with various treatment regimens. (a) Comparison of the percentage of patients experiencing nausea between those treated with conventional TACE and DEB-TACE. (b) Prevalence of nausea in patients treated with TACE loaded with different chemotherapeutic agents. The percentage of patients experiencing nausea varied across different chemotherapeutics, with zinostatin, and adriamycin showing higher rates compared to other agents such as idarubicin and carboplatin. (c) Comparison of the prevalence of nausea in patients undergoing TACE alone versus TACE in combination with other treatments. (d) Prevalence of nausea in patients treated with TACE and adjuvant treatments. The percentage of patients experiencing nausea varied, with patients receiving radiotherapy and 5-fluorouracil showing the highest prevalence, whereas combinations involving mitomycin C or microwave ablation resulted in the lowest prevalence. Bar charts represent mean prevalence ± SEM. DEB-TACE, drug-eluting bead TACE; SEM, standard-error-of-the-mean; TACE, transarterial chemoembolization.

Our meta-analysis identified nausea as a reported side effect in 67 of the studies reviewed (Table 2). The mean prevalence of nausea across these studies was found to be 34.7% (Table 2 and Figure 3(b)). No marked differences were observed between cTACE and DEB-TACE regarding the prevalence of nausea (Figure 5(a)). However, the prevalence of nausea varied depending on the type of chemotherapy used in the TACE emulsion (Figure 5(b)). For example, in the oldest study included in our analyses, 54% of patients reported nausea as an AE after receiving cTACE with zinostatin.^105^ Among the reviewed studies, three used adriamycin within the TACE emulsion, documenting a mean prevalence of nausea at 49.8%.^71,74,91^ The reported prevalence of nausea across these studies varied, ranging from 38% to 65.3%.^71,91^ The lowest prevalence of nausea was reported in studies using carboplatin^9^ and idarubicin^73^ loaded TACE. Combining TACE with other therapies did not significantly affect the prevalence of nausea (Figure 5(c)). The highest prevalence of nausea was reported in one study that combined TACE with radiotherapy and 5-fluorouracil, which led to nausea being reported as AE in 65.3% of patients^91^ (Figure 5(d)). The lowest prevalence of nausea in TACE with different combinational treatments was reported in a study combining TACE with microwave ablation, with only 4.5% of patients reporting nausea as AE.^43^

### Abdominal pain

**Figure 6.**
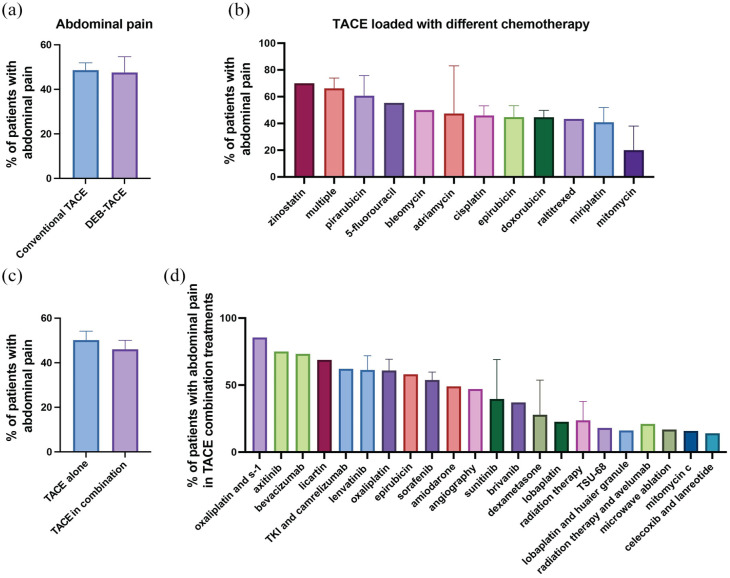
Prevalence of abdominal pain in patients undergoing TACE with various treatment regimens. (a) Comparison of the percentage of patients experiencing abdominal pain between those treated with conventional TACE and DEB-TACE. (b) Prevalence of abdominal pain in patients treated with TACE loaded with different chemotherapeutic agents. The percentage of patients experiencing abdominal pain varied across different chemotherapeutics, with studies using zinostatin and pirarubicin reporting higher prevalences than studies using mitomycin and miriplatin. (c) Comparison of the prevalence of abdominal pain in patients undergoing TACE alone versus TACE in combination with adjuvant treatments. (d) Prevalence of abdominal pain in patients treated with TACE in combination with various adjuvant treatments. The percentage of patients experiencing abdominal pain varied, with oxaliplatin and S1 showing the highest prevalence, whereas combinations involving celecoxib and lanreotide resulted in the lowest prevalence. Bar charts represent mean prevalence ± SEM. DEB-TACE, drug-eluting bead TACE; SEM, standard-error-of-the-mean; TACE, transarterial chemoembolization.

Our meta-analysis revealed that abdominal pain was reported as a side effect in 59 of the studies examined (Table 2). The mean prevalence of abdominal pain across these studies reached 48.1% (Table 2 and Figure 3(b)). We observed no substantial differences in the prevalence of abdominal pain between patients undergoing cTACE and those treated with DEB-TACE (Figure 6(a)). Nonetheless, the specific type of chemotherapy agent used in the TACE emulsion influenced the prevalence rates (Figure 6(b)). Notably, a study indicated that 70% of patients who received TACE loaded with zinostatin reported abdominal pain as AE,^105^ while the mean prevalence of abdominal pain after receiving mitomycin-loaded TACE was 38% when given alone and only 2% when given in combination with dexametasone.^31,45^ Incorporating additional therapies into the TACE regimen did not markedly change the occurrence rates of abdominal pain (Figure 6(c)). However, abdominal pain was reported by 85.5% of patients treated with TACE in combination with oxaliplatin and S-1^45,66^ and was notably high among those receiving axitinib (75%)^61^ and bevacizumab (73%)^97^ (Figure 6(d)).

### GI toxicity

**Figure 7.**
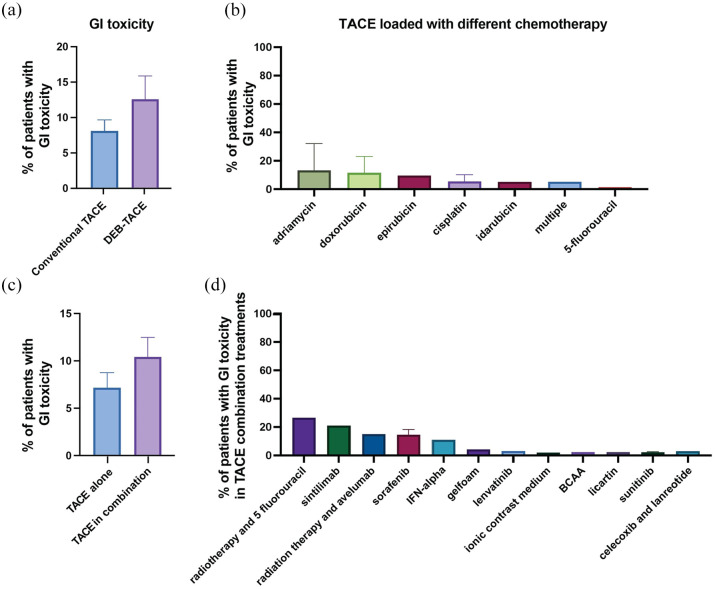
Prevalence of GI toxicity in patients undergoing TACE with various treatment regimens. (a) Comparison of the percentage of patients experiencing GI toxicity between those treated with conventional TACE and DEB-TACE. (b) Prevalence of GI toxicity in patients treated with TACE loaded with different chemotherapeutic agents. The percentage of patients experiencing GI toxicity varied across different chemotherapeutics, with adriamycin and doxorubicin showing higher rates compared to other agents such as 5-fluorouracil. (c) Comparison of the prevalence of GI toxicity in patients undergoing TACE alone versus TACE in combination with adjuvant treatments. (d) Prevalence of GI toxicity in patients treated with TACE in combination with various other treatments. The percentage of patients experiencing GI toxicity varied, with sintilimab and radiotherapy with 5-fluorouracil showing the highest prevalence, whereas combinations involving celecoxib and lanreotide resulted in the lowest prevalence. Bar charts represent mean prevalence ± SEM. DEB-TACE, drug-eluting bead TACE; GI, gastrointestinal; SEM, standard-error-of-the-mean; TACE, transarterial chemoembolization.

Our meta-analysis revealed that GI toxicity was specifically reported as a side effect in 32 of the included studies (Table 2), with an overall mean prevalence of 8.8% (Table 2 and Figure 3(b)). Notably, patients undergoing DEB-TACE exhibited a slightly higher prevalence of GI toxicity than those treated with cTACE (Figure 7(a)). The type of chemotherapy agent used in the TACE emulsion influenced the prevalence rates (Figure 7(b)) with one study reporting 26.6% of patients experiencing GI toxicity following TACE with adriamycin.^91^ However, another study using adriamycin-loaded TACE reported no GI toxicity.^71^ Incorporating additional therapies into the TACE regimen slightly increased the prevalence of GI toxicity (Figure 7(c)). Additionally, incorporating therapies such as radiotherapy and systemic treatment with 5-fluorouracil into the cTACE regimen led to the highest noted prevalence of GI toxicity at 26.6%.^91^ Similarly, treatment with sintilimab prior to receiving DEB-TACE resulted in GI toxicity in 21% in patients, as reported in one study.^33^ The combination of celecoxib and lanreotide had the lowest GI toxicity amongst the adjuvant treatments.^62^

### Overall GI-AEs

**Figure 8.**
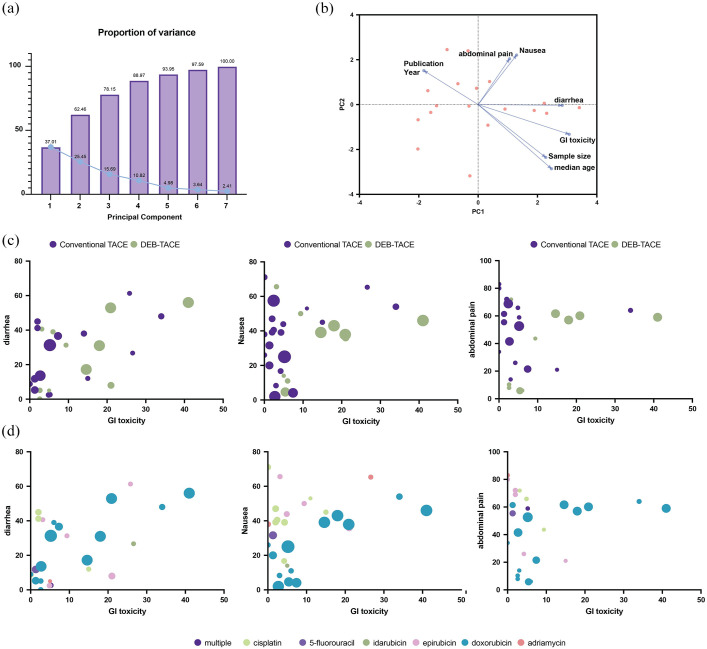
PCA of GI toxicity and other AEs in TACE treatments. (a) Screen plot showing the proportion of variance explained by each principal component. (b) Biplot of the first two principal components showing the relationship between publication year, sample size, median age, and AEs including diarrhea, nausea, abdominal pain, and GI toxicity. (c) Comparison of GI toxicity and diarrhea, nausea, and abdominal prevalence between conventional TACE (purple) and DEB-TACE (green). The size of the dots represents the sample size. (d) Comparison of GI toxicity and diarrhea, nausea, and abdominal prevalence between different chemotherapies used in TACE. The size of the dots represents the sample size of each study arm. AE, adverse effect; DEB-TACE, drug-eluting bead TACE; GI, gastrointestinal; PCA, principal component analysis; TACE, transarterial chemoembolization.

A PCA was performed to reduce the dimensionality of the prevalence data and to identify the primary components contributing to the variance within the dataset (Figure 8(a)). The PCA revealed two principal components that together explained a significant portion of the variance in the symptom prevalences. Principal component 1 (PC1) accounted for a substantial amount of the variance (37.01%) and was primarily associated with direct GI toxicity symptoms. The loadings for PC1 were as follows: GI toxicity (0.863), diarrhea (0.797), nausea (0.364), and abdominal pain (0.297). Principal component 2 (PC2) captured additional 25.46% of variance orthogonal to PC1 and was predominantly associated with nausea and abdominal pain, which are typical features of PES. The loadings for PC2 were nausea (0.852), abdominal pain (0.788), GI toxicity (−0.103), and diarrhea (−0.003). The biplot in Figure 8(b) illustrates the relationships between the studies and the principal components. Studies with high scores on PC1 (right side of the plot) are characterized by elevated prevalence of GI toxicity and diarrhea. This was specifically for one study using doxorubicin-loaded DEB-TACE in combination with sorafenib, which reported 41% of patients experienced GI toxicity and 59% experienced abdominal pain as AE.^60^ Conversely, studies with high scores on PC2 (upper side of the plot) exhibit more prevalence of nausea and abdominal pain as reported side effects. This was specifically notable in two studies, one using cTACE with cisplatin and adjuvant radiotherapy^52^ and another study using epirubicin-loaded DEB-TACE in combination with lenvatinib.^42^ These findings demonstrate that the symptomatology can be effectively summarized along two primary dimensions: one reflecting direct GI toxicity (GI toxicity and diarrhea) and the other reflecting nausea and abdominal pain.

This dimensionality reduction was then used to identify clusters within the dataset, based on the specific treatment modalities (Figure 8(c) and (d)). The scatter plots illustrate the relationship between the prevalence of GI toxicity and diarrhea, nausea, and abdominal pain across the included studies. The size of the dots represents the sample size, while the color distinguishes between DEB-TACE and cTACE in Figure 8(c) and between chemotherapies used within TACE in Figure 8(d). There appears to be a correlation between GI toxicity and diarrhea prevalence, with a higher prevalence of GI toxicity often accompanying higher prevalence in diarrhea, in line with the results of the PCA. No clear clusters were observed in regard to the type of TACE (Figure 8(c)) or the chemotherapy used within TACE (Figure 8(d)) and the prevalence of the different AE.

**Figure 9.**
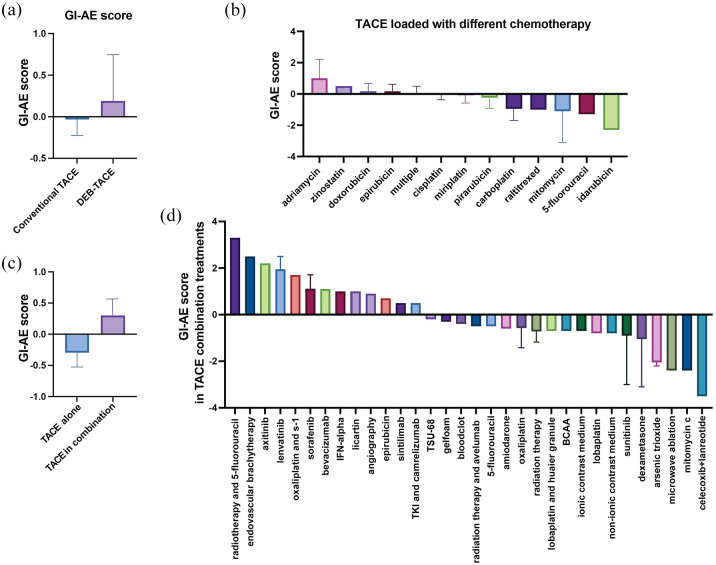
Normalized GI-AE score for patients undergoing TACE with various treatment regimens. (a) Comparison of the GI-AE score between those treated with conventional TACE and DEB-TACE. (b) GI-AE score in patients treated with TACE loaded with different chemotherapeutic agents. The percentage of patients experiencing different GI-AEs varied across different chemotherapeutics, with adriamycin and zinostatin showing a higher score compared to other agents such as 5-fluorouracil and idarubicin. (c) Comparison of GI-AE score in patients undergoing TACE alone versus TACE in combination with adjuvant treatments. (d) GI-AE score in patients treated with TACE in combination with various other treatments. The percentage of patients experiencing different GI-AEs varied, with the combination of radiotherapy with 5-fluorouracil showing the highest overall score, whereas combinations involving celecoxib and lanreotide resulted in the lowest score. Bar charts represent mean prevalence ± SEM. AE, adverse effect; DEB-TACE, drug-eluting bead TACE; GI, gastrointestinal; SEM, standard-error-of-the-mean; TACE, transarterial chemoembolization.

To compare the overall prevalence of all four GI symptoms between the different studies, a normalized GI-AE score was calculated for each study. This showed that cTACE generally led to slightly fewer GI-related AEs than DEB-TACE (Figure 9(a)). The type of chemotherapy agent used in the TACE emulsion influenced the GI-AE score (Figure 9(b)) with adriamycin having a higher overall prevalence of the different GI-AEs^71,74,91^ and idarubicin associated with lower GI-AEs.^73^ Incorporating additional therapies into the TACE regimen slightly increased the GI-AE score (Figure 9(c)). Incorporating therapies such as radiotherapy and systemic treatment with 5-fluorouracil into the cTACE regimen led to the highest overall GI-related side-effects.^91^ The lowest overall GI-related side-effects were noted in a study using celecoxib and lanreotide as adjuvant treatment with cTACE.^62^

## Discussion

This systematic review aimed to map out the prevalence of GI side effects associated with TACE in HCC, with a particular focus on nausea, diarrhea, abdominal pain, and GI toxicity. The findings provide a comprehensive overview of how different chemotherapeutic agents and combination treatments influence these AEs. By treating each arm of these multi-arm RCTs independently, we were able to provide a nuanced understanding of how variations in TACE treatments contribute to the overall landscape of GI toxicity in this patient population.

Certain treatments consistently stood out across different GI side effects. DEB-TACE generally led to slightly higher rates of diarrhea and GI toxicity compared to cTACE.^32,33,42,109^ While some studies suggest that DEB-TACE may offer a safety advantage over cTACE—particularly regarding reduced systemic toxicity and a lower incidence of certain AE—the overall evidence is mixed. Golfieri et al.^110^ noted that while both DEB-TACE and cTACE had similar effectiveness, DEB-TACE was associated with less post-procedural pain. However, results of the PRECISION V randomized trial found that abdominal pain and fatigue occurred more frequently and with greater prevalence in patients receiving DEB-TACE, whereas pyrexia was more common in cTACE patients.^13^ Similarly, a meta-analysis by Wang et al.^111^ revealed no significant difference in the incidence of AEs between DEB-TACE and cTACE. It is important to note that while DEB-TACE is designed to reduce AE by slowly releasing the drug, variations in individual responses and differences in chemotherapy formulations loaded on DEB platforms may influence the outcome. Furthermore, variations in study design, patient populations, and definitions of adverse events make it challenging to draw definitive conclusions.

Globally, DEB-TACE platforms are primarily limited to loading anthracyclines, such as doxorubicin, which is associated with specific side effects including GI toxicity, mucositis, and myelosuppression. Anthracyclines are known to cause GI disturbances like nausea, vomiting, and diarrhea due to their cytotoxic effects on rapidly dividing mucosal cells.^109^ This limitation could contribute to the higher observed rates of certain side effects in DEB-TACE treatments, potentially leading to an overestimation of the AE profile attributed to the DEB-TACE procedure itself. These discrepancies highlight that factors beyond the embolization techniques—such as the specific chemotherapy agents used—may significantly influence the prevalence of GI side effects. Studies that used adriamycin^71,74,91^ reported a higher overall prevalence of the different GI-AE. Notably, the study involving zinostatin reported substantial prevalences of abdominal pain and nausea—70% and 54%, respectively—while only 3% of participants experienced diarrhea.^105^ Although only included in one study, it is important to note that the newer anthracycline idarubicin was associated with a lower overall prevalence of GI-AE.^73^ This is in line with other studies comparing doxorubicin and idarubicin in acute myeloid leukemia.^112^

The findings from our meta-analysis indicate that abdominal pain is the most significant side effect of TACE treatments, with a reported prevalence of 48.1% across 59 studies, followed by nausea, which occurs in 34.7% of patients across 67 studies. This observation aligns with existing literature highlighting the frequent occurrence and clinical relevance of abdominal pain in patients undergoing TACE.^113–115^ Abdominal pain observed in TACE treatments is likely a direct result of the embolization process itself, which can cause ischemia and subsequent pain in the treated liver tissue.^116^ These symptoms are often categorized under PES, which encompasses a range of symptoms—including abdominal pain, fever, nausea, and malaise—that are directly related to the response induced by the embolization of liver tumors. Interestingly, in our PCA, both abdominal pain and nausea clustered together as one principal component (PC2), suggesting that these symptoms are closely linked in their manifestation within PES.

However, it is also essential to consider that abdominal pain can also indicate underlying GI toxicity, a known side effect of certain chemotherapy agents.^16,18,23^ Intestinal toxicity specifically associated with chemotherapy can exacerbate the abdominal discomfort experienced by patients undergoing TACE, highlighting the need for comprehensive management strategies that address both direct procedural effects and systemic chemotherapy-induced toxicity.^16^ Recent attempts have been made to develop a standardized scoring system to predict moderate to severe pain following TACE treatment, which could further improve postoperative pain management and patient outcomes.^113^ Factors such as blood vessel invasion, the use of DEB-TACE, a history of TACE, and prior post-TACE abdominal pain have been shown to be significant predictors of severe pain after TACE treatment,^113,115^ and should be taken into account to provide preventive care. Similarly, Wang et al.^117^ also identified the drug delivery method and the presence of portal vein tumor thrombosis as critical factors for post-TACE pain and PES.

When it comes to drug delivery method, recent studies have explored the efficacy and safety of balloon-occluded TACE (b-TACE) using epirubicin-loaded polyethylene-glycol (PEG) microspheres in patients with HCC.^118–121^ Lucatelli et al.^118^ compared b-TACE with drug-eluting microsphere TACE in 149 patients and found similar oncological responses at most time points. Notably, b-TACE showed a higher objective response rate at 9–12 months and a trend toward longer time to recurrence, with comparable adverse event rates. In another study by Lucatelli et al.,^120^ b-TACE achieved technical success in all procedures among 22 patients with 29 HCC lesions. Adverse events were manageable, with PES occurring in 33% of cases. A further prospective study by Lucatelli et al.^119^ involving 36 patients reported complete response rates over 60% at follow-up intervals up to 18 months using a combined microsphere sizing strategy. AE were minimal, and baseline albumin levels were the only significant predictor of patient outcomes. These findings suggest that b-TACE with epirubicin-loaded PEG microspheres is both effective and safe, potentially offering a favorable GI toxicity profile due to controlled drug release and reduced systemic exposure.^121^

Due to the overall high prevalence of abdominal pain following TACE, effective pain management strategies are essential to improving patient outcomes. Lee et al.^122^ emphasized the efficacy of pre-TACE intra-arterial lidocaine in reducing the prevalence and severity of post-procedural pain. This has led to the recent development of lidocaine/CalliSpheres^®^ composites, which use an electrostatic self-assembly technique to integrate lidocaine with commercial embolic beads.^123^ This approach not only enhances the delivery and localized release of lidocaine during TACE but also significantly reduces post-procedural pain, and modulates the local inflammatory response which could affect the tumor microenvironment.^124–126^ Additionally, Chang et al.^116^ has shown that adjuvant treatment with dexamethasone could significantly reduce TACE-induced AE, including abdominal pain. This has been further established in animal models for chemotherapy-induced intestinal toxicity, suggesting a potential protective effect on the intestinal epithelium.^16,17^ However, it is important to note that while dexamethasone seems to decrease abdominal pain,^31,57^ one study included in our analyses reported that 53.7% of patients who received cTACE in combination with dexamethasone reported nausea as AE.^57^

Our analysis revealed a pattern of selective reporting of GI side effects in the reviewed studies. For instance, nausea was explicitly reported in 83% of the studies, abdominal pain in 73%, diarrhea in 48%, and GI toxicity in 40%. It is important to note that the absence of specific GI side effects in the remaining studies does not necessarily indicate that these side effects were not present; rather, it suggests that these studies did not specifically report on them as outcomes. Importantly, our exclusion criteria were not based on the absence of reports concerning GI toxicity. We excluded studies only if they did not report any GI side effects whatsoever. This variability in symptom reporting likely reflects differences in research objectives, outcome priorities, and the thresholds for AE documentation set by individual researchers.^25^ Considering the relatively high prevalence of certain AE, such as nausea (34.66%), diarrhea (23.46%), and abdominal pain (48.07%), it would be valuable to standardize the reporting of AEs in TACE studies to ensure a more comprehensive understanding of the GI side effect profiles associated with different TACE methodologies.^115,127,128^

There are limitations to consider in this systematic review.^129^ First, we did not exclude studies based on the risk of bias, as we aimed to provide a complete overview of all studies conducted between 2009 and 2023 that met the inclusion criteria. We did perform critical appraisal analyses to ensure that all included studies have sufficient quality to be included in the systematic review.^130,131^ One risk is that some of the studies included had small sample sizes.^35,93,97,101^ From our clustering analyses, it appears that these smaller studies often contributed to an under-reporting of side effects, as studies with larger sample sizes tended to report higher prevalences of side effects (Figure 8(b) and (c)). This suggests that smaller studies might not capture the full spectrum of GI side effects associated with TACE. The variability in reporting specific GI-related AE across studies may also lead to underestimation or overestimation of the actual prevalence rates, complicating the interpretation of data. Information on the extent of the embolization was often missing, which is crucial for understanding the full impact of the procedure. Additionally, this meta-analysis predominantly focuses on immediate and short-term GI AE, with limited data available on long-term complications and outcomes. The lack of detailed information on prophylactic medications administered before and after the TACE procedures in the included studies leaves a gap in our understanding of how these medications might mitigate the side effects associated with TACE. The lack of a standardized practice for TACE, including variations in adjuvant treatments and chemotherapies used within the TACE itself, further complicates the interpretation of results, as a significant portion of this meta-analysis is based on single studies.^130^ However, it is important to recognize that this diversity is also a strength, as it provides a comprehensive overview of the different TACE methodologies and their associated side effects. This broad perspective can be valuable for understanding the range of GI side effects and for identifying potential areas for standardization and improvement in future research.

## Conclusion

In conclusion, our systematic review shows that abdominal pain, nausea, and GI toxicity are common side effects of TACE, and are also influenced by the chemotherapy agents used as well as the inclusion of additional therapies. Effective management protocols are crucial for mitigating these side effects and enhancing the overall well-being of patients undergoing cancer treatment.^132,133^ Standardized reporting of AE in TACE studies would ensure a more comprehensive understanding of the GI side effect profiles associated with different TACE methodologies and can be vital for the quality of life of patients who receive repeated cycles of TACE over a prolonged time period. In addition, there is a need for studies with extended follow-up periods to assess the long-term impact of GI side effects and their influence on patient outcomes.^134^ There is also a need to identify to what extent the different chemotherapies included in TACE induce specific AE, and if there is a specific contribution of the embolization or ischemia itself that could impact AE.^12,135^

## Supplemental Material

sj-docx-1-tam-10.1177_17588359251316663 – Supplemental material for Gastrointestinal side effects in hepatocellular carcinoma patients receiving transarterial chemoembolization: a meta-analysis of 81 studies and 9495 patients Supplemental material, sj-docx-1-tam-10.1177_17588359251316663 for Gastrointestinal side effects in hepatocellular carcinoma patients receiving transarterial chemoembolization: a meta-analysis of 81 studies and 9495 patients by Nathalie Arendt, Maria Kopsida, Jaafar Khaled, Markus Sjöblom and Femke Heindryckx in Therapeutic Advances in Medical Oncology

sj-docx-2-tam-10.1177_17588359251316663 – Supplemental material for Gastrointestinal side effects in hepatocellular carcinoma patients receiving transarterial chemoembolization: a meta-analysis of 81 studies and 9495 patients Supplemental material, sj-docx-2-tam-10.1177_17588359251316663 for Gastrointestinal side effects in hepatocellular carcinoma patients receiving transarterial chemoembolization: a meta-analysis of 81 studies and 9495 patients by Nathalie Arendt, Maria Kopsida, Jaafar Khaled, Markus Sjöblom and Femke Heindryckx in Therapeutic Advances in Medical Oncology

sj-pdf-3-tam-10.1177_17588359251316663 – Supplemental material for Gastrointestinal side effects in hepatocellular carcinoma patients receiving transarterial chemoembolization: a meta-analysis of 81 studies and 9495 patients Supplemental material, sj-pdf-3-tam-10.1177_17588359251316663 for Gastrointestinal side effects in hepatocellular carcinoma patients receiving transarterial chemoembolization: a meta-analysis of 81 studies and 9495 patients by Nathalie Arendt, Maria Kopsida, Jaafar Khaled, Markus Sjöblom and Femke Heindryckx in Therapeutic Advances in Medical Oncology

sj-pdf-4-tam-10.1177_17588359251316663 – Supplemental material for Gastrointestinal side effects in hepatocellular carcinoma patients receiving transarterial chemoembolization: a meta-analysis of 81 studies and 9495 patients Supplemental material, sj-pdf-4-tam-10.1177_17588359251316663 for Gastrointestinal side effects in hepatocellular carcinoma patients receiving transarterial chemoembolization: a meta-analysis of 81 studies and 9495 patients by Nathalie Arendt, Maria Kopsida, Jaafar Khaled, Markus Sjöblom and Femke Heindryckx in Therapeutic Advances in Medical Oncology

sj-pdf-5-tam-10.1177_17588359251316663 – Supplemental material for Gastrointestinal side effects in hepatocellular carcinoma patients receiving transarterial chemoembolization: a meta-analysis of 81 studies and 9495 patients Supplemental material, sj-pdf-5-tam-10.1177_17588359251316663 for Gastrointestinal side effects in hepatocellular carcinoma patients receiving transarterial chemoembolization: a meta-analysis of 81 studies and 9495 patients by Nathalie Arendt, Maria Kopsida, Jaafar Khaled, Markus Sjöblom and Femke Heindryckx in Therapeutic Advances in Medical Oncology

## Declarations

**Ethics approval and consent to participate:** Not applicable.

**Consent for publication:** Not applicable.

**Availability of data and materials:** All data is available as Supplemental Tables.
