# Supplementary material for: Gastrointestinal side effects in hepatocellular carcinoma patients receiving transarterial chemoembolization: a meta-analysis of 81 studies and 9495 patients
Source: Ther Adv Med Oncol. 2025 Feb 7;17:17588359251316663. doi: 10.1177/17588359251316663 (PMC11806495; doi:10.1177/17588359251316663)
Supplement: sj-docx-2-tam-10.1177_17588359251316663 – Supplemental material for Gastrointestinal side effects in hepatocellular carcinoma patients receiving transarterial chemoembolization: a meta-analysis of 81 studies and 9495 patients [file sj-docx-2-tam-10.1177_17588359251316663.docx]

**PubMed** Search: **transarterial chemoembolisation hepatocellular carcinoma**Filters: **Clinical Trial, Randomized Controlled Trial** Sort by: **Most Recent**(("transarterial"[All Fields] OR "transarterially"[All Fields]) AND ("chemoembolic"[All Fields] OR "chemoembolisation"[All Fields] OR "chemoembolisations"[All Fields] OR "chemoembolism"[All Fields] OR "chemoembolization"[All Fields] OR "chemoembolizations"[All Fields] OR "chemoembolized"[All Fields]) AND ("carcinoma, hepatocellular"[MeSH Terms] OR ("carcinoma"[All Fields] AND "hepatocellular"[All Fields]) OR "hepatocellular carcinoma"[All Fields] OR ("hepatocellular"[All Fields] AND "carcinoma"[All Fields]))) AND (clinicaltrial[Filter] OR randomizedcontrolledtrial[Filter])
