# Supplementary material for: Gastrointestinal side effects in hepatocellular carcinoma patients receiving transarterial chemoembolization: a meta-analysis of 81 studies and 9495 patients
Source: Ther Adv Med Oncol. 2025 Feb 7;17:17588359251316663. doi: 10.1177/17588359251316663 (PMC11806495; doi:10.1177/17588359251316663)
Supplement: sj-pdf-3-tam-10.1177_17588359251316663 – Supplemental material for Gastrointestinal side effects in hepatocellular carcinoma patients receiving transarterial chemoembolization: a meta-analysis of 81 studies and 9495 patients [file sj-pdf-3-tam-10.1177_17588359251316663.pdf]

				SCREENING QUESTIONS		2. RANDOMIZED CONTROLLED TRIALS					3. NON-RANDOMIZED STUDIES				
RefID	First author	Year	PMID	S1. Are there clear research questions?	S2. Do the collected data allow to address the research questions?	2.1. Is randomization appropriately performed?	2.2. Are the groups comparable at baseline?	2.3. Are there complete outcome data?	2.4. Are outcome assessors blinded to the intervention provided?	2.5 Did the participants adhere to the assigned intervention?	3.1. Are the participants representative of the target population?	3.2. Are measurements appropriate regarding both the outcome and intervention (or exposure)?	3.3. Are there complete outcome data?	3.4. Are the confounders accounted for in the design and analysis?	3.5. During the study period, is the intervention administered (or exposure occurred) as intended?
1	Li	2023	PMID: 37545497	Yes	Yes						Yes	Yes	Yes	Can't tell	Yes
2	Bush	2023	PMID: 37503907	Yes	Yes						Yes	Yes	Yes	Can't tell	Yes
3	Simasingha	2023	PMID: 36816622	Yes	Yes	Yes	Yes	Yes	Yes	Yes					
4	Shi	2023	PMID: 36794998	Yes	Yes	Yes	Yes	Yes	Yes	Yes					
5	Chengziang	2023	PMID: 36757445	Yes	Yes	Yes	Yes	Yes	Can't tell	Yes					
6	Chiang	2023	PMID: 36529152	Yes	Yes						Yes	Yes	Yes	Can't tell	Yes
7	Comito	2022	PMID: 36421345	Yes	Yes	Yes	Yes	Yes	No	No					
8	Peng	2022	PMID: 35921605	Yes	Yes	Yes	Yes	Yes	No	No					
9	Chen	2022	PMID: 34982369	Yes	Yes	Yes	Yes	Yes	No	No					
10	Li	2022	PMID: 34648352	Yes	Yes	Yes	Can't tell	Yes	No	No					
11	Aramaki	2022	PMID: 34563992	Yes	Yes	Yes	Yes	Yes	No	Yes					
12	Fu	2021	PMID: 34452734	Yes	Yes	Yes	Yes	Yes	No	Yes					
13	Li	2021	PMID: 34269307	Yes	Yes						Yes	Yes	Yes	Can't tell	Yes
14	Ding	2021	PMID: 34237154	Yes	Yes	Yes	Yes	Yes	No	No					
15	Zaltoun	2021	PMID: 33615957	Yes	Yes	Yes	Yes	Yes	Can't tell	Yes					
16	Gjoreski	2021	PMID: 33038087	Yes	Yes	Yes	Yes	Yes	Can't tell	Yes					
17	Guo	2021	PMID: 32774071	Yes	Yes	Yes	Yes	Yes	No	Yes					
18	Turpin	2021	PMID: 32576496	Yes	Yes	Yes	Yes	Yes	Yes	Can't tell					
19	Kudo	2020	PMID: 31801872	Yes	Yes	Yes	Yes	Yes	Yes	Yes					
20	Sun	2020	PMID: 31558180	Yes	Yes						Yes	Yes	Yes	Yes	Can't tell
21	Park	2019	PMID: 30529387	Yes	Yes	Yes	Yes	Yes	Yes	Yes					
22	Zhou	2018	PMID: 29914435	Yes	Yes						Yes	Yes	Yes	Yes	Yes
23	Xu	2018	PMID: 29552783	Yes	Yes	Yes	Yes	Yes	Can't tell	Can't tell					
24	Yoon	2018	PMID: 29543938	Yes	Yes	Yes	Yes	Yes	Yes	Can't tell					
25	Wang	2018	PMID: 29420221	Yes	Yes	Yes	Yes	Yes	No	Can't tell					
26	Zhang	2018	PMID: 29209114	Yes	Yes						Yes	Yes	Yes	Yes	Can't tell
27	He	2017	PMID: 29061175	Yes	Yes						Yes	Yes	Yes	Yes	No
28	Goda	2017	PMID: 28977459	Yes	Yes						Yes	Yes	Yes	Can't tell	No
29	Yang	2017	PMID: 28941589	Yes	Yes	Yes	Yes	Yes	Yes	Can't tell					
30	Ikeda	2018	PMID: 28766016	Yes	Yes	Yes	Yes	Yes	No	No					
31	Zhao GS	2017	PMID: 28723799	Yes	Yes	Yes	Yes	Yes	Can't tell	Yes					
32	Meyer	2017	PMID: 28648803	Yes	Yes	Yes	Yes	Yes	Yes	No					
33	Chan SL	2017	PMID: 28640364	Yes	Yes						Yes	Yes	Yes	Yes	No
34	Tong	2017	PMID: 28430638	Yes	Yes	Yes	Yes	Yes	Yes	Yes					
35	Zhao C	2016	PMID: 27145327	Yes	Yes	Can't tell	Yes	Yes	Can't tell	Yes					
36	Yao X	2016	PMID: 26989044	Yes	Yes						No	Yes	Yes	Yes	Yes
37	Lencioni	2016	PMID: 26809111	Yes	Yes	Yes	Yes	Yes	Yes	No					
38	Ma	2015	PMID: 26625741	Yes	Yes						Yes	Yes	Yes	Can't tell	No
39	Wang	2015	PMID: 26033499	Yes	Yes	Yes	Yes	Yes	Can't tell	Yes					
40	Hoffman	2015	PMID: 25957784	Yes	Yes	Yes	Yes	Yes	Yes	No					
41	Liu B	2015	PMID: 25613214	Yes	Yes	Yes	Yes	Yes	No	Yes					
42	Choi	2014	PMID: 25303890	Yes	Yes						No	Can't tell	Can't tell	Yes	Can't tell
43	Liu B	2015	PMID: 25504506	Yes	Yes	Yes	Yes	Yes	No	Yes					
44	Chao	2015	PMID: 25099027	Yes	Yes						No	Yes	Yes	Can't tell	No
45	El Fouly	2015	PMID: 25040497	Yes	Yes						Yes	Yes	Yes	Can't tell	Can't tell
46	Kudo	2014	PMID: 24996197	Yes	Yes	Yes	Yes	Yes	Yes	No					
47	Boulin	2014	PMID: 24738629	Yes	Yes						Yes	Yes	Yes	Can't tell	Yes
48	Cho	2014	PMID: 24350564	Yes	Yes						No	Yes	Yes	Yes	Can't tell
49	Tzeng	2008	PMID: 18295692	Yes	Yes	Yes	Yes	Yes	Can't tell	Can't tell					
50	Molinari	2006	PMID: 17100154	Yes	Yes						Yes	Yes	Yes	Can't tell	Yes
51	Li	2006	PMID: 17058027	Yes	Yes	Yes	Yes	Can't tell	No	Can't tell					
52	Jang	2004	PMID: 15304127	Yes	Yes	Can't tell	Yes	Yes	Can't tell	Can't tell					
53	Poon	2004	PMID: 15043519	Yes	Yes	Yes	Yes	Yes	No	Yes					
54	Lo	2002	PMID: 11981766	Yes	Yes	Yes	Yes	Yes	No	Yes					
55	Kwok	2000	PMID: 10898316	Yes	Yes	Yes	Yes	Yes	No	Yes					
56	Chung	2000	PMID: 10813709	Yes	Yes	Yes	Yes	Yes	Can't tell	Can't tell					
57	Inaba	2013	PMID: 23764238	Yes	Yes	Can't tell	Yes	Yes	No	No					
58	Kasai	2013	PMID: 23599651	Yes	Yes						Yes	Yes	Yes	Can't tell	Yes
59	Zhai	2013	PMID: 23506690	Yes	Yes	Yes	Yes	Yes	No	Yes					
60	Meyer	2013	PMID: 23449352	Yes	Yes	Yes	Yes	Yes	Can't tell	No					
61	Bai	2013	PMID: 23324079	Yes	Yes						Yes	Yes	Yes	Can't tell	No
62	Iwazawa	2012	PMID: 23155276	Yes	Yes						Yes	Yes	Yes	Can't tell	Can't tell
63	Shi	2013	PMID: 23150720	Yes	Yes	Yes	Yes	Yes	No	No					
64	Chung	2013	PMID: 23129123	Yes	Yes						Yes	Yes	Yes	Can't tell	Can't tell
65	Park	2013	PMID: 23079897	Yes	Yes						Yes	Yes	Yes	Yes	Yes
66	Morimoto	2013	PMID: 23068563	Yes	Yes						Yes	Yes	Yes	Can't tell	Yes
67	Kamimura	2012	PMID: 22994941	Yes	Yes						Yes	Yes	Yes	Can't tell	Can't tell
68	Osuga	2012	PMID: 22922041	Yes	Yes						Yes	Yes	Yes	Can't tell	Yes
69	Sansonno	2012	PMID: 22334456	Yes	Yes	Yes	Yes	Yes	Yes	No					
70	Park	2012	PMID: 22314421	Yes	Yes						Yes	Yes	Yes	Yes	No
71	Britten	2012	PMID: 22244160	Yes	Yes	Yes	Yes	Yes	No	No					
72	Sieghart	2012	PMID: 22215073	Yes	Yes						Yes	Yes	Yes	Can't tell	No
73	Toyame	2012	PMID: 22194039	Yes	Yes						Yes	Yes	Yes	Can't tell	Yes
74	Vogl	2012	PMID: 21940527	Yes	Yes	Yes	Yes	Yes	No	Can't tell					
75	Pawlik	2011	PMID: 21911714	Yes	Yes						Yes	Yes	Yes	Yes	No
76	Boulin	2011	PMID: 21802381	Yes	Yes	Yes	Yes	Yes	No	Yes					
77	Kudo	2011	PMID: 21664811	Yes	Yes	Yes	Yes	Yes	Yes	No					
78	Kim	2011	PMID: 21415578	Yes	Yes						Yes	Yes	Yes	Can't tell	Yes
79	Reyes	2009	PMID: 20010173	Yes	Yes						Yes	Yes	Yes	Can't tell	Yes
80	Okusaka	2009	PMID: 19864035	Yes	Yes	Yes	Yes	Yes	No	No					
81	Romero	2023	PMID: 37037359	Yes	Yes	Yes	Yes	Yes	No	Yes					
