# Supplementary material for: Gastrointestinal side effects in hepatocellular carcinoma patients receiving transarterial chemoembolization: a meta-analysis of 81 studies and 9495 patients
Source: Ther Adv Med Oncol. 2025 Feb 7;17:17588359251316663. doi: 10.1177/17588359251316663 (PMC11806495; doi:10.1177/17588359251316663)
Supplement: sj-pdf-5-tam-10.1177_17588359251316663 – Supplemental material for Gastrointestinal side effects in hepatocellular carcinoma patients receiving transarterial chemoembolization: a meta-analysis of 81 studies and 9495 patients [file sj-pdf-5-tam-10.1177_17588359251316663.pdf]

| Author | Year | Reason |
|---|---|---|
| Savsani E | 2023 | No GI adverse effects reported |
| Guo J, | 2023 | No GI adverse effects reported |
| Goto | 2023 | No GI adverse effects reported |
| Li | 2023 | No GI adverse effects reported |
| Wang | 2023 | No GI adverse effects reported |
| Zhang | 2022 | No GI adverse effects reported |
| Jiang | 2022 | No GI adverse effects reported |
| Yang | 2022 | No GI adverse effects reported |
| Zheng | 2023 | No GI adverse effects reported |
| Bannangkooı | 2022 | No GI adverse effects reported |
| Buckstein | 2022 | Not accessible |
| Liu | 2022 | Not accessible |
| Dhondt | 2022 | No GI adverse effects reported |
| Llovet | 2022 | Type of publication (e.g. protocol, case report) |
| Zhang | 2022 | No GI adverse effects reported |
| Lyu | 2022 | Not accessible |
| Hoshiai | 2022 | Not accessible |
| Tai | 2022 | Not conventional TACE or DEB-TACE |
| Zhang | 2022 | No GI adverse effects reported |
| Ricke | 2021 | Not conventional TACE or DEB-TACE |
| Nutu | 2021 | Not conventional TACE or DEB-TACE |
| Abi-Jauodeh | 2021 | Not conventional TACE or DEB-TACE |
| Bessar | 2021 | Not accessible |
| Zhao | 2021 | Not conventional TACE or DEB-TACE |
| Yi | 2021 | Type of publication (e.g. protocol, case report) |
| Bessar | 2021 | No GI adverse effects reported |
| Yang | 2021 | Not HCC |
| vogi | 2021 | Not HCC |
| Raid | 2020 | Not accessible |
| sebastian | 2020 | Not accessible |
| Sharma | 2020 | No GI adverse effects reported |
| Moche | 2020 | No GI adverse effects reported |
| Thibodeau-aı | 2019 | No GI adverse effects reported |
| Siramolpiwa | 2019 | No GI adverse effects reported |
| Guiu | 2019 | Not accessible |
| Luo | 2019 | Not accessible |
| Chen | 2019 | Type of publication (e.g. protocol, case report) |
| Schnapauff | 2019 | No GI adverse effects reported |
| Mohnike | 2019 | No GI adverse effects reported |
| Wang | 2018 | Language |
| Jiang | 2018 | No GI adverse effects reported |
| Jun | 2018 | No GI adverse effects reported |
| He | 2018 | Not accessible |
| Sato | 2018 | Not accessible |
| Albrecht | 2018 | No GI adverse effects reported |
| de Korompay | 2018 | Not accessible |
| Pinter Carval | 2018 | No GI adverse effects reported |
| Kudo | 2018 | Not conventional TACE or DEB-TACE |
| Mansour | 2018 | No GI adverse effects reported |
| Zhai | 2018 | No GI adverse effects reported |

| | | |
|---|---|---|
| Yao | 2018 | Not accessible |
| Ho yu | 2018 | Not accessible |
| Vilgrain | 2017 | Not conventional TACE or DEB-TACE |
| Habraken | 2017 | Type of publication (e.g. protocol, case report) |
| Hiraoka | 2017 | Not conventional TACE or DEB-TACE |
| Karalli | 2017 | Not hcc |
| Moschouris H | 2017 | No GI adverse effects reported |
| Rodríguez de | 2017 | No GI adverse effects reported |
| Lilienberg E | 2017 | No GI adverse effects reported |
| Negm O, | 2017 | No GI adverse effects reported |
| Salem R | 2016 | No GI adverse effects reported |
| Merchante N | 2017 | Not conventional TACE or DEB-TACE |
| Ippolito D | 2016 | No GI adverse effects reported |
| Chao M | 2016 | No GI adverse effects reported |
| Sheta E | 2016 | No GI adverse effects reported |
| Anota A | 2016 | No GI adverse effects reported |
| Bush | 2016 | No GI adverse effects reported |
| Pokuri | 2018 | No GI adverse effects reported |
| Mohnike | 2016 | Not conventional TACE or DEB-TACE |
| Yu JI | 2016 | Type of publication (Interim analysis) |
| Brown | 2016 | No GI adverse effects reported |
| Liu | 2016 | Type of publication (Retrospective study) |
| Chen CS | 2016 | No GI adverse effects reported |
| Long | 2016 | No GI adverse effects reported |
| Hassan | 2015 | No GI adverse effects reported |
| Hsu | 2016 | Not accessible |
| Wen | 2015 | No GI adverse effects reported |
| Zhang | 2015 | Type of publication (Retrospective study) |
| Pinter | 2015 | Not accessible |
| Breitbach | 2015 | Type of publication (Protocol) |
| Cosgrove | 2015 | Not accessible |
| Kloeckner | 2015 | No GI adverse effects reported |
| Shaw | 2015 | No GI adverse effects reported |
| Wang | 2015 | No GI adverse effects reported |
| Xue | 2015 | No GI adverse effects reported |
| Zhou WZ | 2015 | Not conventional TACE or DEB-TACE |
| Kolligs | 2015 | No GI adverse effects reported |
| Vadot | 2015 | No GI adverse effects reported |
| Pitton | 2015 | No GI adverse effects reported |
| Prajapati | 2014 | Type of publication (Retrospective study) |
| Kim | 2014 | No GI adverse effects reported |
| Corona-Villal | 2014 | No GI adverse effects reported |
| Kloeckner | 2014 | Type of publication (Protocol) |
| Ippolito D | 2014 | No GI adverse effects reported |
| Xu | 2014 | No GI adverse effects reported |
| Ricke | 2015 | Not conventional TACE or DEB-TACE |
| Shina | 2014 | No GI adverse effects reported |
| Zhu | 2014 | Type of publication (Retrospective study) |
| Chen | 2014 | Not conventional TACE or DEB-TACE |
| Guo | 2013 | Not accessible |
| Yamamoto | 2013 | No GI adverse effects reported |

| | | |
|---|---|---|
| Yu | 2014 | No GI adverse effects reported |
| Heibl | 2013 | No GI adverse effects reported |
| Li | 2009 | No GI adverse effects reported |
| Takeshita | 2008 | No GI adverse effects reported |
| Zhou | 2009 | No GI adverse effects reported |
| Zhang | 2009 | No GI adverse effects reported |
| Hoffmann | 2008 | No GI adverse effects reported |
| Graf | 2008 | No GI adverse effects reported |
| Zhang | 2008 | Not accessible |
| Cheng | 2008 | Retracted |
| Doffoël | 2008 | No GI adverse effects reported |
| Yu | 2008 | Not conventional TACE or DEB-TACE |
| Zhou | 2007 | Not accessible |
| Irie | 2007 | No GI adverse effects reported |
| Dharancy | 2007 | No GI adverse effects reported |
| Varela | 2007 | No GI adverse effects reported |
| Li | 2006 | Repeat |
| Dettmer | 2006 | No GI adverse effects reported |
| Jang | 2006 | Not conventional TACE or DEB-TACE |
| Hidajat | 2006 | No GI adverse effects reported |
| Kirchoff | 2006 | Not conventional TACE or DEB-TACE |
| Becker | 2005 | No GI adverse effects reported |
| Gerhards | 2005 | Not conventional TACE or DEB-TACE |
| Liem | 2005 | No GI adverse effects reported |
| Jung | 2005 | No GI adverse effects reported |
| Herber | 2005 | No GI adverse effects reported |
| Kawashima | 2005 | Not conventional TACE or DEB-TACE |
| Jang | 2004 | Not conventional TACE or DEB-TACE |
| Kaibori | 2004 | No GI adverse effects reported |
| Boschi | 2004 | Type of Publication, No GI adverse effects reported |
| Huo | 2004 | No GI adverse effects reported |
| Mazzanti | 2004 | No GI adverse effects reported |
| Huo | 2004 | No GI adverse effects reported |
| Poon | 2004 | Not accessible |
| Vallone | 2003 | Not conventional TACE or DEB-TACE |
| Huo | 2003 | No GI adverse effects reported |
| Wigmore | 2003 | No GI adverse effects reported |
| Yamada | 2003 | No GI adverse effects reported |
| Yuen | 2003 | Not accessible |
| Graziadei | 2003 | No GI adverse effects reported |
| Grieco | 2003 | Not accessible |
| Kim | 2003 | No GI adverse effects reported |
| Roayaie | 2002 | No GI adverse effects reported |
| Lee | 2001 | No GI adverse effects reported |
| Chen | 2001 | Not accessible |
| Gerunda | 2000 | No GI adverse effects reported |
| Alvarez | 2000 | No GI adverse effects reported |
| Cheng | 2000 | No GI adverse effects reported |
| Poon | 1999 | Not accessible |
| Hoshida | 1999 | Not accessible |
| Allgaier | 1999 | No GI adverse effects reported |

| | | |
|---|---|---|
| Bruix | 1998 | Not conventional TACE or DEB-TACE |
| Hasse | 1996 | Not accessible |
| Lee | 1995 | No GI adverse effects reported |
| Shimamura | 1994 | Not accessible |
| Yoshida | 1994 | Not conventional TACE or DEB-TACE |
| Faccioli | 1994 | Not accessible |
| Izumi | 1990 | Not accessible |
| Kim | 2015 | Not conventional TACE or DEB-TACE |
| Takizawa | 2013 | No GI adverse effects reported |
| Iwazawa | 2013 | Type of publication (e.g. protocol, case report) |
| Golfieri | 2013 | Not conventional TACE or DEB-TACE |
| Otto | 2013 | No GI adverse effects reported |
| Shuster | 2013 | No GI adverse effects reported |
| Han | 2013 | Not accessible |
| Bargelini | 2013 | No GI adverse effects reported |
| Wang | 2012 | Not accessible |
| El Kady | 2013 | Not accessible |
| Petruzzi | 2013 | No GI adverse effects reported |
| Prajapati | 2013 | No GI adverse effects reported |
| Kohi | 2013 | No GI adverse effects reported |
| Wiggermanr | 2012 | No GI adverse effects reported |
| Iwazawa | 2012 | No GI adverse effects reported |
| Seinstra | 2012 | No GI adverse effects reported |
| Park | 2012 | No GI adverse effects reported |
| Kang | 2012 | Not conventional TACE or DEB-TACE |
| Bao | 2012 | Not accessible |
| Hoffmann | 2012 | Not conventional TACE or DEB-TACE |
| Yukisawa | 2012 | Not conventional TACE or DEB-TACE |
| Zhao | 2012 | No GI adverse effects reported |
| Cheng | 2012 | Not accessible |
| Uller | 2011 | Not accessible |
| Antoch | 2012 | No GI adverse effects reported |
| Sacco | 2012 | No GI adverse effects reported |
| van Malenste | 2011 | Not accessible |
| Kuwamura | 2011 | No GI adverse effects reported |
| Song | 2011 | No GI adverse effects reported |
| Hoffmann | 2011 | No GI adverse effects reported |
| Kung | 2010 | No GI adverse effects reported |
| Luo | 2011 | Not accessible |
| Bang | 2010 | Not conventional TACE or DEB-TACE |
| Xu | 2010 | Not accessible |
| Dufour | 2010 | No GI adverse effects reported |
| Jin | 2011 | No GI adverse effects reported |
| Chen | 2010 | Not accessible |
| Malagari | 2010 | No GI adverse effects reported |
| Gadaleta | 2009 | No GI adverse effects reported |
| Choi | 2009 | No GI adverse effects reported |
| Palmieri | 2009 | No GI adverse effects reported |
| Poon | 2007 | Not conventional TACE or DEB-TACE |
| Yang | 2014 | No GI adverse effects reported |
| Romero | 2023 | No GI adverse effects reported |
